# Peri-Diagnostic D-Dimer-to-Albumin Ratio and Intensive Care Unit Admission in Pulmonary Embolism: A Single-Center Retrospective Cohort Study

**DOI:** 10.3390/jcm15187107

**Published:** 2026-09-13

**Authors:** Yonghui Wang, Jing Wang, Weihao Li, Xuemin Zhang, Wei Li, Tao Zhang

**Affiliations:** Department of Vascular Surgery, Peking University People’s Hospital, No. 11 Xizhimen South Street, Xicheng District, Beijing 100044, China; 2411110446@stu.pku.edu.cn (Y.W.);

**Keywords:** pulmonary embolism, D-dimer-to-albumin ratio, D-dimer, serum albumin, intensive care unit, risk stratification

## Abstract

**Background/Objectives**: Characterizing factors associated with intensive care unit (ICU) admission among hospitalized patients with pulmonary embolism (PE) is clinically relevant, particularly when established risk-stratification variables are incompletely documented in routine electronic medical records. The D-dimer-to-albumin ratio (DAR) combines a marker of fibrin turnover with serum albumin, a marker of systemic vulnerability; however, whether DAR provides clinically meaningful information beyond D-dimer alone remains uncertain. We evaluated the association between peri-diagnostic DAR and ICU admission and compared DAR with its component biomarkers. **Methods**: This single-center retrospective cohort study used electronic medical record data from 2009 to 2024. Candidate PE-related hospitalizations were identified from diagnostic records and clinically reviewed to verify a current acute PE event. Patients were eligible when DAR were measured within ±48 h of the PE diagnosis timestamp in the electronic medical record; the first eligible hospitalization per patient was retained. The primary outcome was ICU admission. Logistic regression models were adjusted for age, sex, recorded malignancy, and concomitant deep vein thrombosis (DVT). **Results**: Among 159 clinically reviewed patients with current acute PE, 143 (89.9%) had documented objective imaging confirmation, and 22 (13.8%) were admitted to the ICU. Each 1-unit increase in log-DAR was associated with greater odds of ICU admission (odds ratio [OR], 2.02; 95% confidence interval [CI], 1.43–2.86; *p* < 0.001), and the highest DAR quartile (Q4) was associated with greater odds than Q1–Q3 (OR, 6.78; 95% CI, 2.52–18.20; *p* < 0.001). However, in a temporally restricted analysis including 145 patients and 8 subsequent ICU admissions, the associations were attenuated and no longer statistically significant. Log-DAR was almost perfectly correlated with log-D-dimer (Spearman ρ = 0.995) and did not materially improve discrimination (area under the receiver operating characteristic curve [AUC], 0.788 vs. 0.785; ΔAUC, 0.003; *p* = 0.471; optimism-corrected AUC, 0.738 vs. 0.737). **Conclusions**: Peri-diagnostic DAR was associated with ICU admission in the primary analysis, but the temporally restricted analysis did not support prospective prediction of subsequent ICU admission. Its incremental value beyond D-dimer appeared limited, and DAR should be considered an exploratory adjunct rather than a replacement for established PE risk-stratification tools.

## 1. Introduction

Pulmonary embolism (PE) is a major manifestation of venous thromboembolism (VTE) and remains an important cause of clinical deterioration and mortality. In patients with acute PE, management decisions depend not only on confirming the diagnosis but also on early risk stratification based on hemodynamic status, validated clinical scores, right ventricular function, cardiac biomarkers, and imaging findings [1,2]. However, these variables are not always completely or systematically documented in routine electronic medical records. Readily available laboratory parameters may therefore provide complementary information regarding the clinical status of hospitalized patients with PE, although they cannot replace established risk-stratification approaches.

D-dimer is widely used in the diagnostic evaluation of suspected PE. Beyond its diagnostic role, higher D-dimer concentrations have been associated with hypotension, tachycardia, hypoxemia, greater pulmonary arterial obstruction, right ventricular dilatation, and myocardial injury in patients with acute PE [3,4]. Nevertheless, D-dimer has limited specificity and may be influenced by age, malignancy, infection, inflammation, surgery, and other clinical conditions. Serum albumin is another routinely measured laboratory parameter that reflects nutritional status, systemic inflammation, vascular permeability, and overall physiological reserve. Previous studies have shown that hypoalbuminemia is associated with adverse short-term outcomes in patients with acute PE and may provide additional prognostic information when combined with established clinical risk scores [5,6].

The D-dimer-to-albumin ratio (DAR) combines a marker of fibrin turnover with a marker of systemic vulnerability into a simple composite index. DAR has been investigated for the identification of preoperative deep vein thrombosis (DVT) in older patients with hip fracture and for risk assessment in patients with PE associated with hematologic malignancies [7,8,9]. However, evidence regarding DAR in broader populations of hospitalized patients with PE remains limited. Moreover, because D-dimer varies over several orders of magnitude whereas serum albumin generally varies within a relatively narrow range, the association between DAR and clinical outcomes may be driven predominantly by the D-dimer component. Whether DAR provides meaningful information beyond D-dimer and serum albumin considered separately therefore remains uncertain.

In this study, we evaluated the association between peri-diagnostic DAR and intensive care unit admission in a retrospective cohort of hospitalized patients with PE. Respiratory support and vasoactive drug use were assessed as exploratory outcomes. We also compared DAR with D-dimer and serum albumin to assess its incremental model performance beyond its component biomarkers.

## 2. Methods

### 2.1. Study Design and Data Source

This single-center retrospective cohort study used data from the electronic medical record system of Peking University People’s Hospital from 2009 through 2024. The database contained structured and unstructured clinical information, including patient demographics, encounter and diagnostic records, laboratory measurements, vital signs, medication and non-medication orders, intensive care records, death records, imaging reports, and ultrasound reports. All patient-level data were de-identified before analysis.

The primary objective was to evaluate the association between the peri-diagnostic D-dimer-to-albumin ratio (DAR) and intensive care unit (ICU) admission during hospitalization among patients with pulmonary embolism (PE). Respiratory support and vasoactive drug use were evaluated as exploratory outcomes. This study is reported in accordance with the Strengthening the Reporting of Observational Studies in Epidemiology (STROBE) recommendations.

The study protocol was approved by the Ethics Committee of Peking University People’s Hospital (approval No. 2025PHB509-001). The requirement for informed consent was waived because of the retrospective study design and the use of de-identified data. The study was conducted in accordance with the Declaration of Helsinki.

### 2.2. Study Population

PE-related encounters were initially identified from the diagnosis database using terms including “pulmonary embolism”, “pulmonary arterial embolism”, “pulmonary thromboembolism”, or equivalent Chinese terms. Diagnostic-term extraction was used for candidate identification only. Before final inclusion, available clinical records were reviewed at the case level to verify a current acute PE event based on available in-hospital or documented external imaging/diagnostic evidence, compatible clinical presentation, and PE-directed management. Historical, chronic, residual/follow-up, or suspected but unconfirmed PE alone was not considered sufficient for inclusion. Incidentally detected PE was retained when imaging supported a current acute event.

The cohort was restricted to hospitalized encounters with paired D-dimer and serum albumin measurements obtained within ±48 h of the recorded PE diagnosis. Neither a conventional nor an age-adjusted D-dimer threshold was used for cohort identification or eligibility. This symmetric window was used because the recorded diagnosis timestamp may not coincide exactly with clinical recognition or initial diagnostic testing; the measurements were therefore considered peri-diagnostic rather than strictly baseline values. If multiple measurements were available, the earliest eligible measurements were selected. For patients with more than one eligible hospitalization, only the first was retained in the primary analysis.

### 2.3. Exposure Variable

The primary exposure variable was DAR, calculated as follows: DAR = D-dimer/serum albumin. All analyzed D-dimer and serum albumin values were expressed on common scales of ng/mL and g/L, respectively, before DAR calculation. Reference-range normalization or conversion between fibrinogen-equivalent units (FEU) and D-dimer units (DDU) was not performed because assay designation, detection limits, and temporal changes in analyzers or reagents were not consistently available; medication and procedure records also did not reliably establish actual treatment administration or the timing of anticoagulation, systemic thrombolysis, or catheter-directed therapy relative to biomarker sampling. Because DAR had a right-skewed distribution, natural-log-transformed DAR (log-DAR) was used as the primary continuous exposure. DAR quartiles were defined according to the distribution in the primary analysis cohort. Q4 versus Q1–Q3 was evaluated as a secondary, exploratory, and data-derived contrast to examine whether the association was concentrated among patients with the highest DAR values. In this cohort, Q4 corresponded to DAR values ≥ 73.55. This comparison was not prespecified and does not represent a clinically validated threshold.

### 2.4. Outcomes

The primary outcome was ICU admission during the index hospitalization. ICU admission was identified using intensive care records and relevant hospitalization records. Because a PE-specific indication could not be reliably adjudicated from the retrospective records, ICU admission was treated as an all-cause critical-care resource-use outcome rather than a PE-specific severity endpoint. Secondary and exploratory outcomes included respiratory support, vasoactive drug use, and in-hospital death. Respiratory support included invasive mechanical ventilation, non-invasive ventilation, or high-flow oxygen therapy when identifiable from medical records. Vasoactive drug use was identified from ICU medication records and medication orders. In-hospital death was identified from death records. All outcomes were restricted to events occurring during the index hospitalization.

### 2.5. Covariates and Additional Variables

Given the limited number of ICU admission events, the primary adjusted model was deliberately restricted to four covariates selected on the basis of clinical relevance and data availability: age, sex, recorded malignancy, and concomitant DVT. No automated or outcome-driven variable-selection procedure was used, and additional covariates were evaluated separately in sensitivity analyses rather than entered simultaneously. Recorded malignancy was defined as the presence of any malignant neoplasm diagnosis in the structured diagnostic records used for cohort construction. The available diagnostic data did not reliably distinguish active cancer from a remote history of malignancy. Concomitant DVT was defined as the presence of DVT in the diagnostic records during the same PE-related encounter. Vital signs included heart rate and systolic blood pressure. The shock index was calculated as heart rate divided by systolic blood pressure. Hemoglobin, platelet count, and serum creatinine were used for descriptive and univariable analyses; other conditions affecting D-dimer or albumin, including hepatic or renal disease, infection or inflammation, nutritional status, recent surgery, and fluid balance, were not systematically available for multivariable adjustment. The data sources, definitions, adjudication rules, and availability of key variables are summarized in Supplementary Table S1.

### 2.6. Statistical Analysis

Continuous variables are presented as mean ± standard deviation or median with interquartile range, as appropriate. Categorical variables are presented as counts and percentages. Baseline characteristics and clinical outcomes were compared across DAR quartiles. Continuous variables were compared using analysis of variance or the Kruskal–Wallis test, depending on distribution. Categorical variables were compared using the chi-square test or Fisher’s exact test. To assess potential selection related to laboratory availability, hospitalized candidate PE encounters with and without eligible paired measurements were compared using available demographic and clinical characteristics, with absolute standardized mean differences (SMDs) additionally reported.

Logistic regression was used to assess the association between DAR and ICU admission. Univariable logistic regression was first performed to evaluate the associations of log-DAR, DAR quartiles, DAR Q4 versus Q1-Q3, component biomarkers, and clinical variables with ICU admission. Multivariable logistic regression models were then constructed. Model 1 was unadjusted. Model 2 was adjusted for age and sex. Model 3 was further adjusted for recorded malignancy and concomitant DVT. Results are reported as odds ratios (ORs) with 95% confidence intervals (CIs). Given the limited number of outcome events, Firth’s bias-reduced penalized logistic regression was additionally performed as a sensitivity analysis for the primary ICU models, shock-index-adjusted models, and sparse exploratory outcome models. Penalized profile-likelihood 95% CIs were calculated.

To evaluate whether DAR provided incremental information beyond its component biomarkers, a base model including age, sex, recorded malignancy, and concomitant DVT was compared with models additionally containing log-D-dimer, log-albumin, log-DAR, or log-D-dimer and log-albumin entered as separate variables. Albumin was natural-log-transformed in this comparison because log-DAR is mathematically equivalent to log-D-dimer minus log-albumin. Model discrimination and fit were assessed using the area under the receiver operating characteristic curve (AUC) with DeLong 95% confidence intervals and the Akaike information criterion (AIC), respectively. Changes in AUC were assessed using paired DeLong tests. Corresponding receiver operating characteristic (ROC) curves were plotted for the four biomarker models. The nested log-D-dimer model and log-D-dimer-plus-log-albumin model were additionally compared using a likelihood-ratio test. Internal validation with 2000 nonparametric bootstrap resamples was used to estimate optimism-corrected AUCs, calibration intercepts, and calibration slopes. Net reclassification analysis was not performed because of the limited number of outcome events and the absence of prespecified clinically relevant risk thresholds.

To visually assess the relationship between continuous log-DAR and ICU admission, an adjusted logistic fitted curve was generated based on the primary adjusted model. An exploratory restricted cubic spline model adjusted for age, sex, recorded malignancy, and concomitant DVT was fitted using four knots at the 5th, 35th, 65th, and 95th percentiles of log-DAR (−0.123, 2.151, 3.811, and 5.704), with the median log-DAR value (2.984) as the reference. Overall association and nonlinearity were assessed using Wald χ^2^ tests.

### 2.7. Sensitivity and Exploratory Analyses

Several sensitivity analyses were performed to evaluate the robustness of the findings. First, all primary-model variables were complete, whereas additional adjustment for shock index used 106 complete cases; no imputation was performed, and missingness was not assumed to be completely at random. A temporally restricted analysis was performed by excluding patients whose selected DAR measurement occurred at or after the first ICU admission. For temporal classification, the reference time for DAR was defined as the later of the selected D-dimer and serum albumin collection times, because both measurements were required to calculate the ratio. Historical ICU records with date-only timestamps were additionally reviewed using contemporaneous ICU-transfer or critical-care orders. A separate diagnosis-time-restricted analysis included only patients whose selected D-dimer and serum albumin measurements were both obtained at or before the recorded PE diagnosis. Because only three ICU admission events remained, this analysis was performed using Firth-penalized logistic regression with the same adjustment variables. Second, the analyses were repeated after excluding patients with recorded malignancy and after excluding the highest 1% of D-dimer values. Third, the outcome was separately changed to respiratory support and vasoactive drug use to evaluate the association between DAR and other indicators of critical care resource use.

In exploratory supplementary analyses, the availability and distribution of imaging and echocardiographic features related to PE severity were summarized descriptively, including central PE, pulmonary infarction, right heart strain, and elevated pulmonary artery pressure. Patients without relevant imaging or echocardiographic reports were classified as having unavailable data rather than as having no abnormality. Because these variables were incompletely and non-systematically available, no multivariable imaging-adjusted model was fitted.

All statistical tests were two-sided. Effect estimates and 95% CIs were emphasized, and *p* values were not interpreted as the sole criterion for evidence. Conventional analyses were performed using R version 4.2.3, whereas the Firth analyses were implemented in Python version 3.12 using Jeffreys-prior penalized likelihood.

## 3. Results

### 3.1. Study Population and Baseline Characteristics

**Figure 1:**
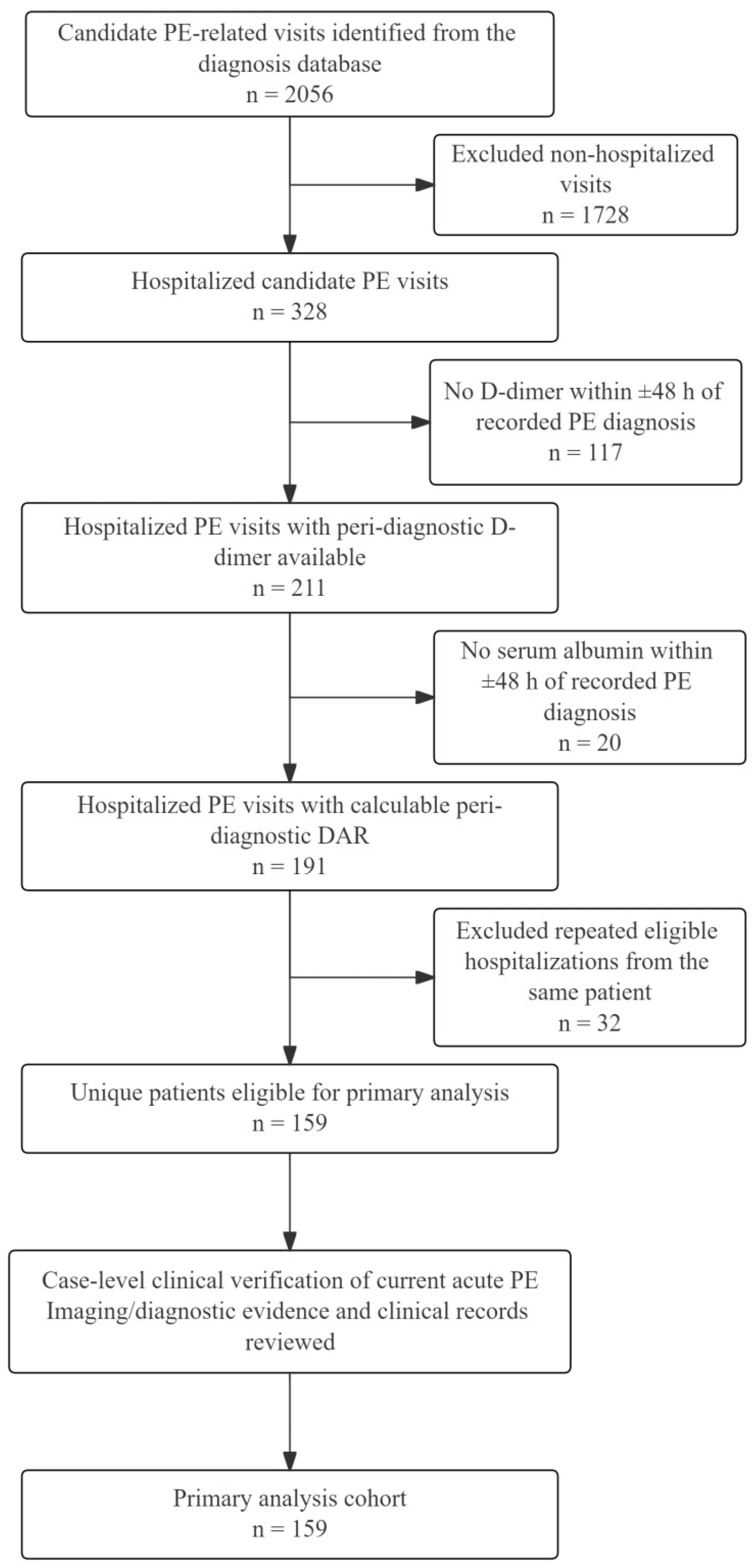
Flowchart of study cohort identification and selection. PE-related visits were identified from the diagnosis database. After restricting to hospitalized PE visits, excluding visits without eligible D-dimer or serum albumin measurements, and retaining only the first eligible hospitalization per patient, 159 patients were included in the primary analysis, of whom 22 were admitted to the ICU during the index hospitalization. DAR, D-dimer-to-albumin ratio; PE, pulmonary embolism.

Between 2009 and 2024, 2056 candidate PE-related encounters were identified from the diagnosis database. Of these, 1728 were non-hospitalized encounters, leaving 328 hospitalized candidate PE encounters. Among the hospitalized encounters, 117 had no D-dimer measurement within ±48 h of the recorded PE diagnosis, and 20 of the remaining 211 had no serum albumin measurement within the same window, yielding 191 hospitalizations with calculable peri-diagnostic DAR. After retaining only the first eligible hospitalization per patient, 159 patients were included in the primary analysis. Case-level clinical review supported a current acute PE event in all 159 patients. Objective imaging confirmation was documented in 143 patients (89.9%), including 112 with evidence available from our institution and 31 with documented external imaging confirmation. In the remaining 16 patients (10.1%), the original external imaging was unavailable for independent re-review, but acute PE was supported by documented external diagnosis and compatible clinical records. The 159 patients were divided into DAR quartiles: Q1, Q2, Q3, and Q4 included 40, 40, 40, and 39 patients, respectively (Figure 1).

Overall, the mean age was 58.8 ± 16.4 years, and 78 patients (49.1%) were male. There were no statistically significant differences across DAR quartiles in age, sex, admission year, recorded malignancy, concomitant DVT, hemoglobin, platelet count, serum creatinine, systolic blood pressure, or shock index. Consistent with the mathematical construction of DAR, D-dimer concentrations increased and serum albumin concentrations decreased across DAR quartiles (both *p* < 0.001). Heart rate showed a modest difference across DAR quartiles (*p* = 0.048), whereas no clear between-group differences were observed in the other reported clinical characteristics (Table 1).

To assess potential selection related to laboratory availability, the 191 hospitalized encounters with eligible paired measurements were compared with the 137 hospitalized encounters without eligible paired measurements (Supplementary Table S2). Age, sex, and recorded malignancy were broadly similar between groups, whereas laboratory-eligible encounters occurred later in the study period and had higher frequencies of concomitant DVT and ICU admission. In-hospital deaths occurred only among laboratory-ineligible encounters.

### 3.2. Clinical Outcomes

**Figure 2:**
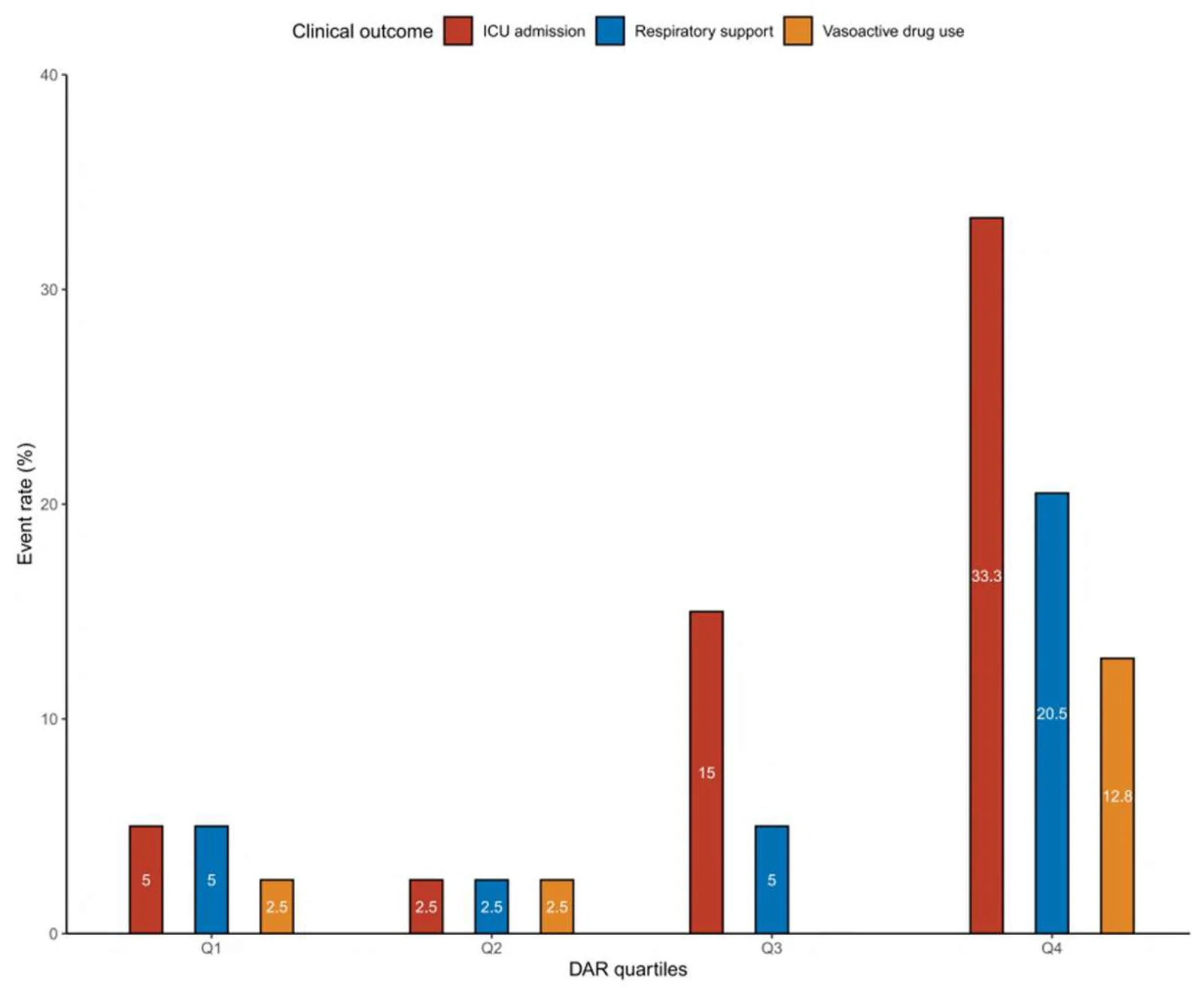
Clinical outcome rates across DAR quartiles in the primary analysis cohort (*n* = 159)**.** The numbers of events were 22 for ICU admission, 13 for respiratory support, and 7 for vasoactive drug use. DAR, D-dimer-to-albumin ratio; ICU, intensive care unit.

During the index hospitalization, 22 patients (13.8%) were admitted to the ICU. Three of these 22 patients were already in the ICU at the time of the recorded PE diagnosis. ICU admission rates were 5.0%, 2.5%, 15.0%, and 33.3% across Q1–Q4, respectively (*p* < 0.001). Respiratory support was required in 13 patients (8.2%), and vasoactive drugs were used in 7 patients (4.4%). Both exploratory outcomes were numerically most frequent in Q4, with corresponding rates of 20.5% and 12.8% (*p* = 0.023 and p = 0.047, respectively). No in-hospital deaths occurred in the primary analysis cohort (Table 2 and Figure 2).

D-dimer and serum albumin had the same recorded collection time in 144 of 159 patients (90.6%), with a median interval of 0 h (IQR, 0–0 h). Among the 22 ICU admissions, the selected peri-diagnostic DAR preceded the first ICU admission in 8 patients (36.4%) and was obtained at or after ICU admission in 14 patients (63.6%); three patients were already in the ICU when PE was recorded. ICU indications were PE-related compromise or management in 12 patients, peri-procedural VTE monitoring in 3, non-PE postoperative or other illness in 4, and mixed or indeterminate in 3.

### 3.3. Association Between DAR and ICU Admission

In univariable logistic regression analysis, each 1-unit increase in log-DAR was associated with 93% greater odds of ICU admission (OR, 1.93; 95% CI, 1.39–2.68; *p* < 0.001). Patients in Q4 had greater odds of ICU admission than those in Q1–Q3 (OR, 6.17; 95% CI, 2.38–15.96; *p* < 0.001). Among the component biomarkers, log-D-dimer showed a similar magnitude of association with ICU admission, whereas higher serum albumin was associated with lower odds of ICU admission. Age, sex, recorded malignancy, concomitant DVT, systolic blood pressure, and shock index were not clearly associated with ICU admission in the univariable analyses (Table 3).

**Figure 3:**
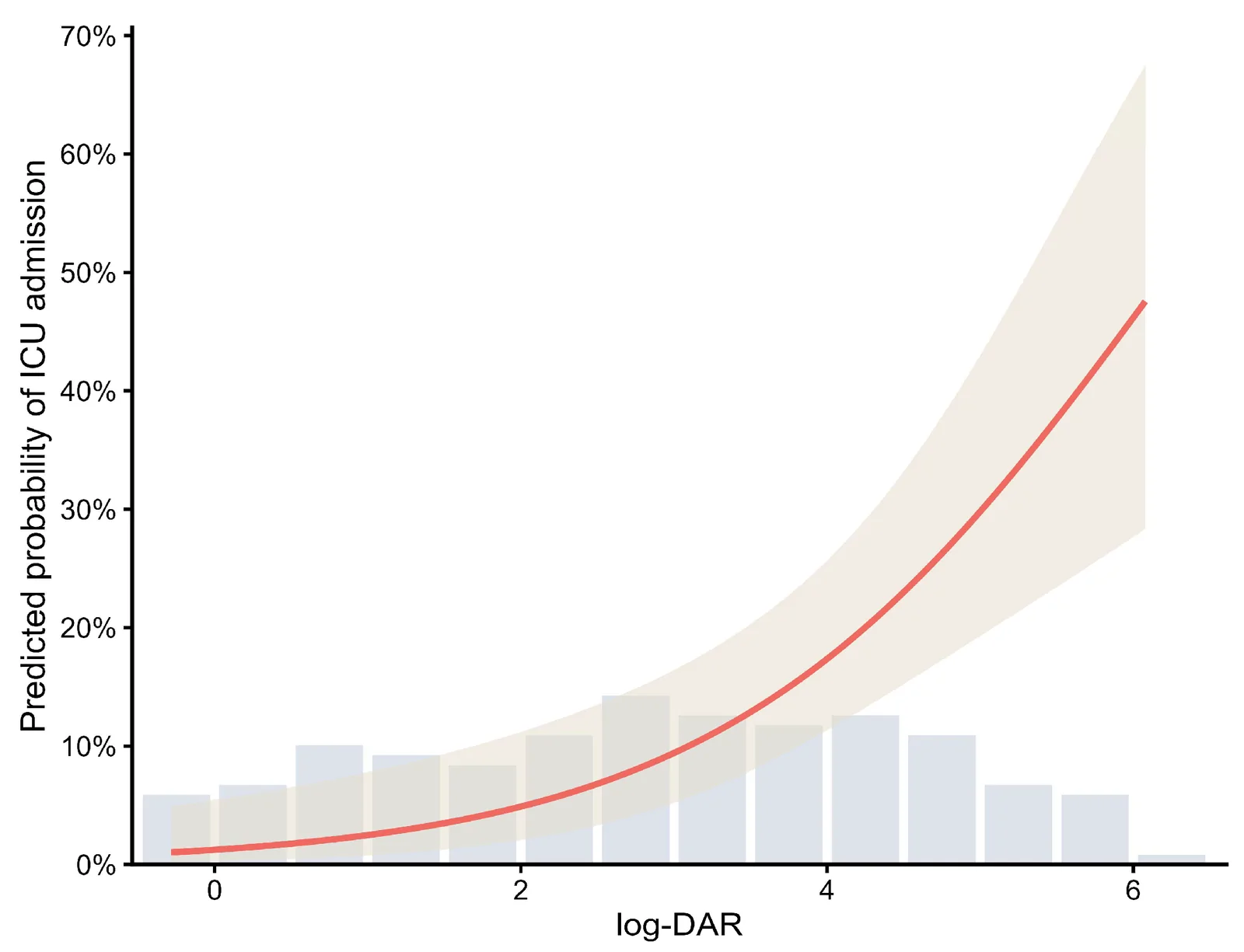
Adjusted logistic fitted curve for the association between log-DAR and ICU admission. The analysis included 159 patients and 22 ICU admission events. The curve shows the adjusted model-estimated probability of ICU admission across log-DAR values. The model was adjusted for age, sex, recorded malignancy, and concomitant DVT, and the shaded area represents the 95% CI. CI, confidence interval; DAR, D-dimer-to-albumin ratio; DVT, deep vein thrombosis; ICU, intensive care unit.

The magnitude and direction of the association were similar across the unadjusted and multivariable-adjusted models. In Model 3, each 1-unit increase in log-DAR was associated with approximately two-fold greater odds of ICU admission (OR, 2.02; 95% CI, 1.43–2.86; *p* < 0.001). Patients in Q4 also had greater odds of ICU admission than those in Q1–Q3 (OR, 6.78; 95% CI, 2.52–18.20; *p* < 0.001) (Table 4). The adjusted logistic fitted curve showed an increasing model-estimated probability of ICU admission across higher log-DAR values (Figure 3). The exploratory restricted cubic spline analysis showed an overall association between log-DAR and ICU admission (*p* for overall association = 0.001), without evidence of nonlinearity (*p* for nonlinearity = 0.907; Supplementary Figure S1).

### 3.4. Results of Sensitivity and Exploratory Analyses

**Figure 4:**
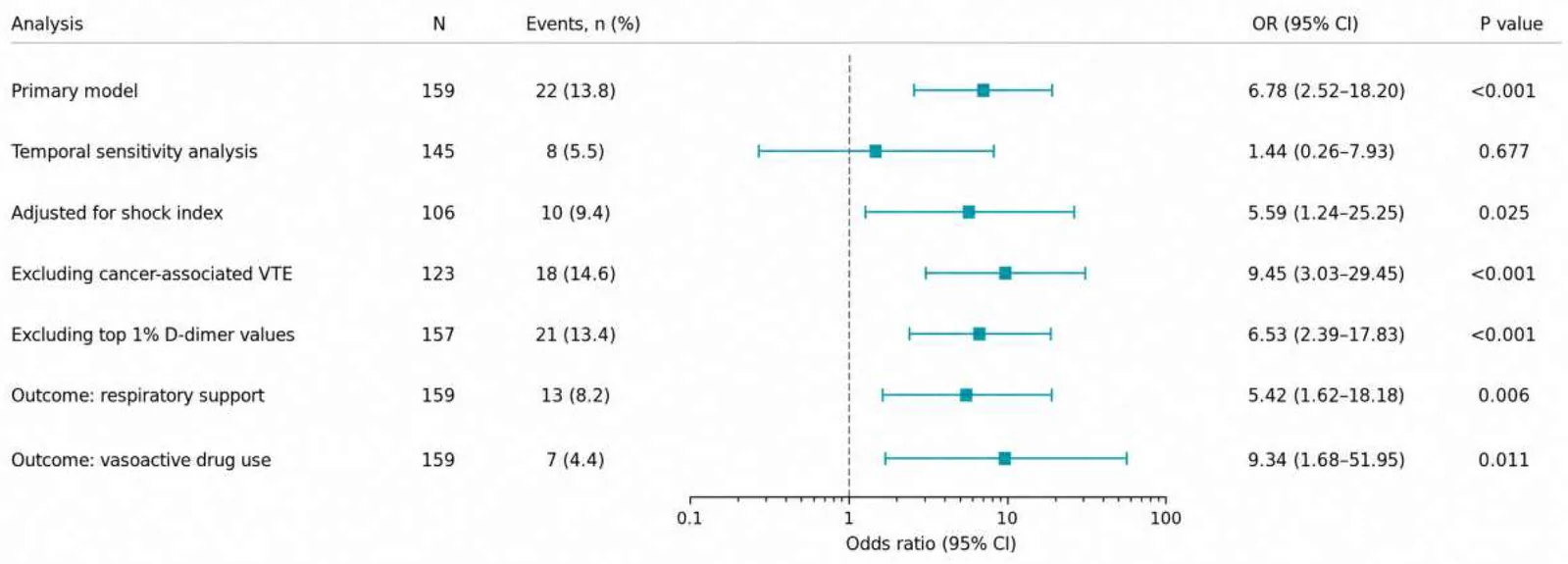
Sensitivity and exploratory analyses of DAR and clinical outcomes. Analysis-specific sample sizes and outcome-event counts were as follows: primary ICU model, 159 patients/22 events; shock-index-adjusted model, 106 patients/10 events; exclusion of recorded malignancy, 123 patients/18 events; exclusion of the highest 1% of D-dimer values, 157 patients/21 events; temporally restricted cohort, 145 patients/8 subsequent ICU events; respiratory support, 159 patients/13 events; and vasoactive drug use, 159 patients/7 events. CI, confidence interval; DAR, D-dimer-to-albumin ratio; ICU, intensive care unit; OR, odds ratio; VTE, venous thromboembolism.

The results remained directionally consistent after additional adjustment for shock index (106 complete cases; 10 ICU admissions; shock index missing in 12 of 22 ICU-admitted patients and 41 of 137 patients without ICU admission), exclusion of patients with recorded malignancy, and exclusion of D-dimer values above the 99th percentile (Table 5 and Figure 4). By contrast, in the temporally restricted cohort comprising 145 patients and 8 subsequent ICU admissions, the associations were attenuated and imprecise. The adjusted ORs were 1.29 (95% CI, 0.84–1.97) per 1-unit increase in log-DAR and 1.44 (95% CI, 0.26–7.93) for Q4 versus Q1–Q3. In the separate diagnosis-time-restricted analysis, 29 patients and 3 ICU admission events remained. Firth penalized estimates were imprecise: the adjusted OR was 1.53 (95% profile-likelihood CI, 0.75–6.25; *p* = 0.262) per 1-unit increase in log-DAR and 1.89 (95% profile-likelihood CI, 0.15–18.88; *p* = 0.587) for DAR Q4 versus Q1–Q3 (Table 5).

Associations with respiratory support and vasoactive drug use were directionally consistent with those for ICU admission but were based on only 13 and 7 events, respectively, and remained imprecise. Firth penalized logistic regression yielded slightly attenuated but directionally consistent estimates for ICU admission: 1.92 (95% profile-likelihood CI, 1.41–2.75) per 1-unit increase in log-DAR and 6.12 (95% profile-likelihood CI, 2.41–16.24) for Q4 versus Q1–Q3. Estimates from the shock-index-adjusted and sparse exploratory outcome models were similarly imprecise (Supplementary Table S3).

Patients in the primary analysis cohort were hospitalized between 2012 and 2024. ICU admission occurred in 10 of 78 patients (12.8%) during 2012–2021 and 12 of 81 patients (14.8%) during 2022–2024. After additional adjustment for calendar year of admission, both conventional and Firth estimates remained similar to the primary results. In the Firth models, the adjusted OR was 1.95 (95% profile-likelihood CI, 1.43–2.83) per 1-unit increase in log-DAR and 6.26 (95% profile-likelihood CI, 2.45–16.87) for DAR Q4 versus Q1–Q3 (Supplementary Table S3).

PE-related imaging and echocardiographic reports containing sufficient information for extraction of specific severity features were available for 58 and 45 patients, respectively. The availability and distribution of extractable findings are summarized descriptively in Supplementary Table S4. Patients without relevant reports were classified as having unavailable data rather than negative findings. Because of the substantial missingness, non-systematic imaging availability, and small number of outcome events, no multivariable imaging-adjusted analysis was performed.

### 3.5. Comparison with Component Biomarkers

Log-DAR was almost perfectly correlated with log-D-dimer (Spearman ρ = 0.995), consistent with the substantially greater variability of log-D-dimer than log-albumin on the log scale (standard deviation [SD], 1.78 vs. 0.16). The model containing log-D-dimer yielded an apparent AUC of 0.785 (95% CI, 0.682–0.888), an optimism-corrected AUC of 0.737, and an AIC of 118.4. The corresponding values were 0.743 (95% CI, 0.638–0.847), 0.660, and 132.6 for log-albumin; 0.792 (95% CI, 0.697–0.887), 0.738, and 119.0 for log-D-dimer and log-albumin entered separately; and 0.788 (95% CI, 0.687–0.889), 0.738, and 117.5 for log-DAR. Compared with the log-D-dimer model, the change in AUC was 0.003 for log-DAR (DeLong *p* = 0.471) and 0.008 for the model containing log-D-dimer and log-albumin separately (DeLong *p* = 0.486). Adding log-albumin to the log-D-dimer model did not significantly improve model fit (likelihood-ratio χ^2^ = 1.42; degrees of freedom [df] = 1; *p* = 0.234). Optimism-corrected calibration slopes were 0.770 for log-D-dimer, 0.723 for log-D-dimer plus log-albumin, and 0.762 for log-DAR. These findings provided no evidence of clinically meaningful incremental performance from forming DAR or adding log-albumin to log-D-dimer (Supplementary Table S5 and Supplementary Figure S2).

## 4. Discussion

In this single-center retrospective cohort of hospitalized patients with PE, higher peri-diagnostic DAR was associated with greater odds of ICU admission in the primary analysis. However, the selected DAR preceded the first ICU admission in only 8 of 22 ICU-admitted patients, and the association was substantially attenuated in the temporally restricted analysis. The primary finding should therefore be interpreted as a contemporaneous association with critical-care resource use, rather than as evidence that DAR directly or independently reflects PE severity or prospectively predicts subsequent ICU admission. Associations in the same direction were also observed for respiratory support and vasoactive drug use, although these exploratory analyses were based on few events. Importantly, log-DAR was highly correlated with log-D-dimer and showed similar model performance; therefore, the findings do not demonstrate improved risk prediction beyond D-dimer alone.

Current PE risk assessment integrates hemodynamic status, validated clinical tools such as the Pulmonary Embolism Severity Index and its simplified version, right ventricular function, cardiac biomarkers, and imaging findings [2,10,11,12]. Clinical criteria such as the Hestia rule are also used to identify selected patients who may be suitable for outpatient management, while guideline-based management decisions remain anchored in established clinical and physiological indicators [13,14]. The present study was not designed to develop or validate a new prediction score or to replace these established approaches. Rather, it evaluated whether a routinely available laboratory ratio was associated with ICU admission in an electronic medical record cohort in which several standard severity variables were incompletely documented.

The close correspondence between DAR and D-dimer warrants cautious interpretation. In the present cohort, log-DAR was almost perfectly correlated with log-D-dimer, and the two models showed nearly identical apparent and optimism-corrected discrimination. Neither formation of DAR nor addition of log-albumin to the log-D-dimer model significantly improved AUC or model fit. Bootstrap correction also demonstrated calibration slopes below 1 for all biomarker models, consistent with some degree of overfitting in this small cohort. These findings suggest that the observed DAR–ICU association was driven largely by its D-dimer component and do not establish meaningful incremental predictive value beyond D-dimer alone.

Serum albumin may nevertheless provide clinically relevant information that is not fully captured by D-dimer. Hypoalbuminemia may reflect inflammation, malnutrition, altered vascular permeability, endothelial dysfunction, and reduced physiological reserve [15,16,17,18]. Albumin contributes to the maintenance of oncotic pressure, endothelial glycocalyx integrity, and microcirculatory homeostasis; however, a low measured concentration may simultaneously reflect inflammation-related capillary leakage, altered distribution, and underlying disease severity [17,18]. A small randomized study in hypoalbuminemic ICU patients reported fewer and less severe pressure ulcers with albumin administration, providing a non-PE clinical example of tissue vulnerability [19]. Accordingly, the present association should be interpreted primarily as a marker of systemic vulnerability rather than evidence that hypoalbuminemia is a PE-specific causal mechanism or a modifiable therapeutic target. Lower serum albumin has also been associated with venous thromboembolism and adverse short-term outcomes in patients with acute PE [5,6,20]. In the present study, higher serum albumin was associated with lower odds of ICU admission in the univariable analysis. However, neither combining albumin with D-dimer in the same model nor forming DAR materially improved model performance. Previous studies of DAR have focused mainly on preoperative deep vein thrombosis in older patients with hip fracture and on PE associated with hematologic malignancies [7,8,9]. Our findings extend the evaluation of DAR to a hospitalized PE cohort outside these previously studied settings, but support its interpretation as a compact composite of two routinely available biomarkers rather than as a biomarker with demonstrated superiority over D-dimer.

Several established PE severity variables, including oxygenation, the Pulmonary Embolism Severity Index (PESI) and the simplified Pulmonary Embolism Severity Index (sPESI), cardiac biomarkers, standardized right ventricular assessment, and thrombus burden [21,22,23,24], were not systematically available, precluding comprehensive adjustment for conventional PE risk stratification. Additional adjustment for shock index provided only a partial sensitivity assessment. Imaging and echocardiographic information was too incomplete and non-systematically available for reliable multivariable adjustment and was therefore summarized descriptively only. Residual confounding by established PE severity markers consequently cannot be excluded, and the observed DAR–ICU association should not be interpreted as evidence of independent predictive value beyond these markers.

Although the adjusted DAR model showed an AUC of 0.788 (optimism-corrected AUC, 0.738) and an optimism-corrected calibration slope of 0.762, these derivation-cohort performance measures and the statistically significant ORs do not establish clinical utility. DAR did not materially improve discrimination over D-dimer alone, and net clinical benefit was not evaluated. Because complete PESI/sPESI components and other established PE severity markers were unavailable, direct comparison with validated PE risk-stratification tools was not possible. The findings therefore do not support the use of DAR for prospective ICU triage or treatment decisions; it should be regarded only as an exploratory descriptive adjunct. External validation, comparison with established PE scores, and assessment of calibration and decision-curve net benefit are required before clinical application [2,10,11,12,13,14].

This study has several limitations. First, the single-center design, small sample size, and only 22 ICU admission events relative to the number of model parameters increased the risk of overfitting and unstable or exaggerated estimates, despite restricting the primary adjustment set and performing Firth penalized sensitivity analyses. the unexpected univariable associations of higher hemoglobin and lower platelet count with ICU admission may reflect chance, residual confounding, or selected case mix and should not be interpreted causally. Selection bias related to laboratory availability and the complete-case shock-index analysis is also possible, as shock-index missingness differed by ICU status and hospitalized encounters with and without eligible paired measurements differed particularly in admission year, ICU admission, and in-hospital mortality; the high prevalence of concomitant DVT may additionally reflect the referral pattern and case mix of a tertiary vascular-surgery population. Although all included cases were clinically adjudicated as current acute PE, original external imaging was unavailable for independent re-review in some patients. Second, the predefined ±48 h laboratory window did not ensure that DAR preceded ICU admission; only eight subsequent ICU events remained in the temporally restricted analysis, limiting precision and precluding a prospective predictive interpretation. Within this window, serum albumin and D-dimer concentrations may also have been influenced by unmeasured hepatic or renal disease, infection or inflammation, nutritional status, recent surgery, fluid administration, hemodilution, or treatment initiated before sampling; treatment-related confounding and residual temporal assay heterogeneity therefore cannot be excluded. Third, ICU admission is a critical-care resource-use outcome influenced by institutional practice, bed availability, clinician decision-making, monitoring requirements, comorbid illness, and temporal changes in practice; therefore, its association with DAR should not be interpreted as evidence that DAR directly or independently reflects PE severity. Fourth, no in-hospital deaths occurred in the primary analytic cohort, precluding evaluation of the association between DAR and mortality. Fifth, several established PE risk-stratification variables, including oxygenation, cardiac biomarkers, standardized right ventricular assessment, thrombus burden, and complete PESI/sPESI components, were not systematically available, leaving residual confounding by PE severity. Finally, because DAR was highly correlated with D-dimer, its incremental value beyond D-dimer and established risk-stratification tools requires confirmation in larger multicenter prospective cohorts.

## 5. Conclusions

In this retrospective cohort of hospitalized patients with clinically reviewed acute PE, higher DAR was associated with ICU admission during the index hospitalization. However, DAR was highly correlated with D-dimer and showed limited evidence of incremental discrimination or model fit beyond D-dimer alone. This study does not establish that DAR prospectively predicts future ICU admission or identify a clinically applicable DAR threshold for ICU triage or treatment decisions. These findings should therefore be considered hypothesis-generating, and DAR should be regarded as an exploratory adjunct rather than a stand-alone clinical decision-making tool.

## Figures and Tables

**Table 1:** Baseline characteristics according to D-dimer-to-albumin ratio quartiles.

Characteristic	Overall (*n* = 159)	Q1 (*n* = 40)	Q2 (*n* = 40)	Q3 (*n* = 40)	Q4 (*n* = 39)	*p* Value
Age, years	58.8 ± 16.4	55.6 ± 15.7	62.7 ± 15.9	55.8 ± 17.2	61.0 ± 16.4	0.125
Male sex, *n* (%)	78 (49.1)	16 (40.0)	19 (47.5)	21 (52.5)	22 (56.4)	0.510
Admission year	2022 (2019, 2023)	2022 (2019, 2023)	2022 (2019, 2023)	2022 (2020, 2023)	2021 (2019, 2024)	0.974
Recorded malignancy, *n* (%)	36 (22.6)	9 (22.5)	13 (32.5)	7 (17.5)	7 (17.9)	0.376
Concomitant DVT, *n* (%)	134 (84.3)	33 (82.5)	32 (80.0)	34 (85.0)	35 (89.7)	0.703
D-dimer, ng/mL	743.0 (156.5, 2669.5)	75.5 (37.8, 96.0)	415.5 (274.5, 541.5)	1436.5 (973.8, 1901.0)	5394.0 (3289.5, 9375.5)	<0.001
Serum albumin, g/L	37.9 ± 5.3	39.8 ± 4.3	40.0 ± 3.6	38.2 ± 4.6	33.6 ± 5.9	<0.001
DAR	19.8 (4.1, 71.7)	1.9 (1.0, 2.6)	9.9 (6.7, 14.2)	42.5 (25.7, 52.5)	150.3 (106.9, 289.9)	<0.001
Hemoglobin, g/L	126.8 ± 22.1	129.8 ± 12.6	126.8 ± 21.0	126.9 ± 29.5	123.4 ± 22.7	0.650
Platelet count, ×10^9^/L	209.0 (165.8, 260.2)	204.5 (176.2, 250.0)	217.0 (156.5, 279.5)	240.0 (180.5, 287.0)	203.5 (133.5, 225.0)	0.190
Creatinine, μmol/L	67.0 (59.5, 86.0)	63.5 (56.0, 73.2)	72.0 (59.0, 90.2)	65.5 (62.0, 80.8)	70.0 (62.5, 88.5)	0.097
Heart rate, beats/min	80.3 ± 11.8	76.4 ± 8.9	78.3 ± 9.2	82.4 ± 10.8	84.5 ± 16.2	0.048
SBP, mmHg	132.1 ± 20.1	133.0 ± 20.5	133.6 ± 17.8	130.6 ± 20.6	131.2 ± 21.9	0.904
Shock index	0.59 (0.54, 0.68)	0.58 (0.50, 0.63)	0.57 (0.54, 0.63)	0.63 (0.53, 0.71)	0.63 (0.54, 0.74)	0.102

Data are presented as mean ± SD, median (IQR), or *n* (%), as appropriate. DAR, D-dimer-to-albumin ratio; DVT, deep vein thrombosis; IQR, interquartile range; SBP, systolic blood pressure; SD, standard deviation.

**Table 2:** Clinical outcomes according to DAR quartiles.

Outcome	Overall (*n* = 159)	Q1 (*n* = 40)	Q2 (*n* = 40)	Q3 (*n* = 40)	Q4 (*n* = 39)	*p* Value
ICU admission	22 (13.8)	2 (5.0)	1 (2.5)	6 (15.0)	13 (33.3)	<0.001
Respiratory support	13 (8.2)	2 (5.0)	1 (2.5)	2 (5.0)	8 (20.5)	0.023
Vasoactive drug use	7 (4.4)	1 (2.5)	1 (2.5)	0 (0.0)	5 (12.8)	0.047
In-hospital death	0 (0.0)	0 (0.0)	0 (0.0)	0 (0.0)	0 (0.0)	—

Data are presented as *n* (%). DAR, D-dimer-to-albumin ratio; ICU, intensive care unit. Respiratory support included invasive or non-invasive ventilatory support or high-flow oxygen therapy when recorded.

**Table 3:** Univariable logistic regression analysis for ICU admission.

Variable	OR (95% CI)	*p* Value
DAR-related variables		
Log-DAR, per 1-unit increase	1.93 (1.39–2.68)	<0.001
DAR Q4 vs. Q1–Q3	6.17 (2.38–15.96)	<0.001
DAR quartile Q2 vs. Q1	0.49 (0.04–5.60)	0.564
DAR quartile Q3 vs. Q1	3.35 (0.63–17.74)	0.155
DAR quartile Q4 vs. Q1	9.50 (1.98–45.67)	0.005
Component biomarkers		
Log-D-dimer, per 1-unit increase	1.93 (1.38–2.71)	<0.001
Serum albumin, per 1 g/L increase	0.88 (0.80–0.95)	0.003
Clinical characteristics		
Age, per 1-year increase	1.00 (0.98–1.03)	0.865
Male sex	0.85 (0.34–2.09)	0.716
recorded malignancy	0.73 (0.23–2.31)	0.591
Concomitant DVT	0.81 (0.25–2.65)	0.733
Vital signs		
Heart rate, per 10 beats/min increase	1.15 (0.70–1.91)	0.580
SBP, per 10 mmHg increase	0.99 (0.79–1.25)	0.944
Shock index, per 0.1 increase	0.98 (0.57–1.71)	0.951
Additional laboratory variables		
Hemoglobin, per 10 g/L increase	1.27 (1.03–1.57)	0.026
Platelet count, per 50 × 10^9^/L increase	0.48 (0.33–0.70)	<0.001
Creatinine, per 10 μmol/L increase	0.99 (0.85–1.16)	0.931

Odds ratios were estimated using univariable logistic regression. CI, confidence interval; DAR, D-dimer-to-albumin ratio; DVT, deep vein thrombosis; ICU, intensive care unit; OR, odds ratio; SBP, systolic blood pressure.

**Table 4:** Multivariable logistic regression analysis of DAR and ICU admission.

Model	Adjustment	log-DAR, per 1-Unit Increase OR (95% CI)	log-DAR *p* Value	DAR Q4 vs. Q1–Q3 OR (95% CI)	DAR Q4 vs. Q1–Q3 *p* Value
Model 1	Unadjusted	1.93 (1.39–2.68)	<0.001	6.17 (2.38–15.96)	<0.001
Model 2	Age and sex	1.98 (1.41–2.77)	<0.001	6.50 (2.47–17.11)	<0.001
Model 3	Age, sex, recorded malignancy, and concomitant DVT	2.02 (1.43–2.86)	<0.001	6.78 (2.52–18.20)	<0.001

Model 1 was unadjusted. Model 2 was adjusted for age and sex. Model 3 was adjusted for age, sex, recorded malignancy, and concomitant DVT. OR for log-DAR represents the effect per 1-unit increase. CI, confidence interval; DAR, D-dimer-to-albumin ratio; DVT, deep vein thrombosis; ICU, intensive care unit; OR, odds ratio.

**Table 5:** Sensitivity analyses for the association between DAR and clinical outcomes.

Analysis	Outcome	Exposure	*N*	Events	OR (95% CI)	*p* Value
Primary model	ICU admission	log-DAR, per 1-unit increase	159	22	2.02 (1.43–2.86)	<0.001
Primary model	ICU admission	DAR Q4 vs. Q1–Q3	159	22	6.78 (2.52–18.20)	<0.001
Additionally adjusted for shock index	ICU admission	log-DAR, per 1-unit increase	106	10	1.89 (1.16–3.10)	0.011
Additionally adjusted for shock index	ICU admission	DAR Q4 vs. Q1–Q3	106	10	5.59 (1.24–25.25)	0.025
Excluding recorded malignancy	ICU admission	log-DAR, per 1-unit increase	123	18	2.18 (1.45–3.27)	<0.001
Excluding recorded malignancy	ICU admission	DAR Q4 vs. Q1–Q3	123	18	9.45 (3.03–29.45)	<0.001
Excluding top 1% D-dimer values	ICU admission	log-DAR, per 1-unit increase	157	21	2.09 (1.43–3.05)	<0.001
Excluding top 1% D-dimer values	ICU admission	DAR Q4 vs. Q1–Q3	157	21	6.53 (2.39–17.83)	<0.001
Outcome changed to respiratory support	Respiratory support	log-DAR, per 1-unit increase	159	13	1.66 (1.14–2.42)	0.009
Outcome changed to respiratory support	Respiratory support	DAR Q4 vs. Q1–Q3	159	13	5.42 (1.62–18.18)	0.006
Outcome changed to vasoactive drug use	Vasoactive drug use	log-DAR, per 1-unit increase	159	7	1.98 (1.15–3.41)	0.014
Outcome changed to vasoactive drug use	Vasoactive drug use	DAR Q4 vs. Q1–Q3	159	7	9.34 (1.68–51.95)	0.011
Temporal sensitivity analysis	Subsequent ICU admission	log-DAR	145	8	1.29 (0.84–1.97)	0.249
Temporal sensitivity analysis	Subsequent ICU admission	DAR Q4 vs. Q1–Q3	145	8	1.44 (0.26–7.93)	0.677
Diagnosis-time-restricted analysis (Firth penalized)	ICU admission	log-DAR, per 1-unit increase	29	3	1.53 (0.75–6.25)	0.262
Diagnosis-time-restricted analysis (Firth penalized)	ICU admission	DAR Q4 vs. Q1–Q3	29	3	1.89 (0.15–18.88)	0.587

Models were adjusted for age, sex, recorded malignancy, and concomitant DVT unless otherwise specified. In the temporal sensitivity analysis, non-ICU patients were retained and ICU patients were included only when DAR was measured before the first ICU admission. The original DAR quartile classification was retained. CI, confidence interval; DAR, D-dimer-to-albumin ratio; DVT, deep vein thrombosis; ICU, intensive care unit; OR, odds ratio. In the diagnosis-time-restricted analysis, both selected biomarkers were obtained at or before the recorded PE diagnosis. Because only three ICU events remained, Firth-penalized logistic regression adjusted for age, sex, recorded malignancy, and concomitant DVT was used, with profile penalized-likelihood 95% CIs; the quartile categories defined in the primary cohort were retained.

## Data Availability

The datasets analyzed during the current study are not publicly available because of patient privacy and institutional data protection requirements. Anonymized data may be made available from the corresponding author upon reasonable request and with permission from Peking University People’s Hospital, subject to applicable ethical and institutional restrictions.

## Supplementary Materials

The following supporting information can be downloaded at: https://www.mdpi.com/article/10.3390/jcm15187107/s1, Table S1: Definitions and data availability of key study variables; Table S2: Comparison of hospitalized candidate PE encounters with and without eligible paired peri-diagnostic D-dimer and serum albumin measurements; Table S3: Comparison of conventional and Firth penalized logistic regression estimates for DAR and clinical outcomes; Table S4: Availability and descriptive distribution of imaging and echocardiographic findings according to DAR quartiles; Table S5: Correlations, model performance, internal validation, and formal comparisons of DAR and its component biomarkers for ICU admission. Figure S1: Restricted cubic spline analysis of log-DAR and ICU admission; Figure S2: ROC curves comparing DAR and its component biomarkers for ICU admission.
