# Supplementary material for: Peri-Diagnostic D-Dimer-to-Albumin Ratio and Intensive Care Unit Admission in Pulmonary Embolism: A Single-Center Retrospective Cohort Study
Source: J Clin Med. 2026 Sep 13;15(18):7107. doi: 10.3390/jcm15187107 (PMC13607606; doi:10.3390/jcm15187107)
Supplement: Supplementary file 1 [file jcm-15-07107-s001.zip › jcm-4508220-supplementary.pdf]

Supplementary tables and figures

Supplementary Table S1. Definitions and data availability of key study variables

Variable	Definition	Source	Time window / rule	Data available / positive, n (%)
D-dimer	Plasma D-dimer concentration.	Laboratory database	The earliest available value within ±48 hours of PE diagnosis was used.	159 (100.0)
Serum albumin	Serum albumin concentration.	Laboratory database	The earliest available value within ±48 hours of PE diagnosis was used.	159 (100.0)
D-dimer-to-albumin ratio (DAR)	Ratio of plasma D-dimer to serum albumin.	Derived from laboratory variables	Calculated when both D-dimer and serum albumin were available within ±48 hours of PE diagnosis.	159 (100.0)
DAR quartiles	Quartile categories of DAR in the primary analysis cohort.	Derived variable	Q1–Q4 were defined according to the distribution of DAR in the primary analysis cohort.	159 (100.0)
ICU admission	Admission to an intensive care unit during the index hospitalization.	ICU records	Any ICU admission record during the index hospitalization.	159 (100.0)
Respiratory support	Use of invasive mechanical ventilation, non-invasive ventilation, or high-flow oxygen therapy.	Non-drug orders / ICU records	Any recorded respiratory support during the index hospitalization.	159 (100.0)
Vasoactive drug use	Use of recorded vasoactive agents during the index hospitalization.	Medication records / ICU medication records	Any recorded vasoactive drug administration during the index hospitalization.	159 (100.0)
In-hospital death	Death during the index hospitalization.	Death records	Any in-hospital death record.	159 (100.0)
Recorded malignancy	Any malignant neoplasm diagnosis recorded during or before the index hospitalization.	Diagnosis database	Identified from available diagnosis records; active and historical malignancy could not be reliably distinguished.	159 (100.0)

Variable	Definition	Source	Time window / rule	Data available / positive, n (%)
Concomitant DVT	Deep vein thrombosis recorded during the same PE-related hospitalization or visit.	Diagnosis database	Identified from diagnosis records of the index visit.	159 (100.0)
Heart rate	Heart rate recorded during the early assessment period.	Vital signs database	The earliest available value in the early assessment period was used.	106 (66.7)
Systolic blood pressure	Systolic blood pressure recorded during the early assessment period.	Vital signs database	The earliest available value in the early assessment period was used.	154 (96.9)
Shock index	Heart rate divided by systolic blood pressure.	Derived from vital signs	Calculated using the earliest available heart rate and systolic blood pressure in the early assessment period.	106 (66.7)
PE severity-feature imaging report availability	Availability of a PE-related imaging report containing sufficient detail for extraction of prespecified severity features.	Imaging reports	Extracted from available imaging report text.	58 (36.5)
Central PE	Pulmonary embolism involving the pulmonary trunk, main pulmonary arteries, or pulmonary artery bifurcation.	Imaging reports	Identified from available PE-related imaging reports using sentence-level text extraction to reduce misclassification from negated or historical descriptions.	6 (3.8)
Pulmonary infarction	Imaging report indicating pulmonary infarction or suspected pulmonary infarction.	Imaging reports	Extracted from available PE-related imaging report text.	2 (1.3)
Echocardiography availability	Availability of echocardiographic information during the index hospitalization.	Ultrasound reports	Extracted from available ultrasound report text.	45 (28.3)
Right heart strain	Echocardiographic evidence suggestive of right-sided cardiac enlargement, right ventricular overload, or	Ultrasound reports	Extracted from available echocardiographic report text.	8 (5.0)

Variable	Definition	Source	Time window / rule	Data available / positive, n (%)
	reduced right ventricular function.			
Elevated pulmonary artery pressure	Echocardiographic report indicating elevated pulmonary artery pressure or numeric PASP ≥40 mmHg.	Ultrasound reports	Extracted from available echocardiographic text or structured numeric results.	8 (5.0)
PASP	Estimated pulmonary artery systolic pressure on echocardiography.	Ultrasound reports	Numeric value extracted from echocardiographic text or structured result when available.	25 (15.7)

For clinical outcomes and diagnosis-based covariates, variables were considered ascertained for all patients in the primary analysis cohort. For imaging and echocardiographic variables, the last column indicates the number and percentage of patients with available or positive findings derived from non-structured clinical reports. DAR, D-dimer-to-albumin ratio; DVT, deep vein thrombosis; ICU, intensive care unit; PASP, pulmonary artery systolic pressure; PE, pulmonary embolism; VTE, venous thromboembolism.

Supplementary Table S2. Comparison of hospitalized candidate PE encounters with and without eligible paired peri-diagnostic D-dimer and serum albumin measurements.

Characteristic	Eligible paired measurements (n = 191)	No eligible paired measurements (n = 137)	p Value	SMD
Age, years	57.7 ± 16.5	59.9 ± 16.3	0.230	0.134
Male sex, n (%)	92 (48.2)	74 (54.0)	0.351	0.117
Admission year, median (IQR)	2022 (2019–2023)	2017 (2012–2023)	<0.001	0.839
recorded malignancy, n (%)	41 (21.5)	31 (22.6)	0.908	0.028
Concomitant DVT, n (%)	165 (86.4)	108 (78.8)	0.098	0.200
ICU admission, n (%)	22 (11.5)	3 (2.2)	0.001	0.376
In-hospital death, n (%)	0 (0.0)	7 (5.1)	0.002	0.328

Values are presented as mean ± standard deviation, median (interquartile range), or n (%), as appropriate. P values were obtained using Welch's t-test, Wilcoxon rank-sum test, chi-square test, or Fisher's exact test, as appropriate. Absolute standardized mean differences are reported. DVT, deep

vein thrombosis; ICU, intensive care unit; PE, pulmonary embolism; SMD, standardized mean difference; VTE, venous thromboembolism.

Supplementary Table S3. Comparison of conventional and Firth penalized logistic regression estimates for DAR and clinical outcomes.

Analysis	Outcome	Exposure	N	Events	Conventional logistic OR (95% CI)	Firth penalized OR (95% profile-likelihood CI)
Primary model	ICU admission	log-DAR, per 1-unit increase	159	22	2.02 (1.43–2.86)	1.92 (1.41–2.75)
Primary model	ICU admission	DAR Q4 vs Q1–Q3	159	22	6.78 (2.52–18.20)	6.12 (2.41–16.24)
Additionally adjusted for admission year	ICU admission	log-DAR, per 1-unit increase	159	22	2.08 (1.45–2.98)	1.95 (1.43–2.83)
Additionally adjusted for admission year	ICU admission	DAR Q4 vs Q1–Q3	159	22	7.06 (2.58–19.33)	6.26 (2.45–16.87)
Additionally adjusted for shock index	ICU admission	log-DAR, per 1-unit increase	106	10	1.89 (1.16–3.10)	1.73 (1.14–2.84)
Additionally adjusted for shock index	ICU admission	DAR Q4 vs Q1–Q3	106	10	5.59 (1.24–25.25)	4.66 (1.18–19.77)
Exploratory alternative outcome	Respiratory support	log-DAR, per 1-unit increase	159	13	1.66 (1.14–2.42)	1.59 (1.14–2.35)
Exploratory alternative outcome	Respiratory support	DAR Q4 vs Q1–Q3	159	13	5.42 (1.62–18.18)	4.91 (1.59–16.25)
Exploratory alternative	Vasoactive drug use	log-DAR, per 1-unit	159	7	1.98 (1.15–3.41)	1.81 (1.15–3.16)

Analysis	Outcome	Exposure	N	Events	Conventional logistic OR (95% CI)	Firth penalized OR (95% profile-likelihood CI)
outcome		increase				
Exploratory alternative outcome	Vasoactive drug use	DAR Q4 vs Q1–Q3	159	7	9.34 (1.68–51.95)	7.29 (1.70–41.09)

Values are presented as odds ratios with 95% confidence intervals. Conventional logistic regression estimates were obtained using maximum likelihood estimation with Wald confidence intervals. Firth estimates were obtained using bias-reduced penalized-likelihood logistic regression with profile penalized-likelihood confidence intervals. All models were adjusted for age, sex, recorded malignancy, and concomitant DVT. Models additionally adjusted for admission year included calendar year of admission as a continuous covariate. Models additionally adjusted for shock index included shock index as an additional covariate and were based on complete cases. Analyses of calendar year, respiratory support, and vasoactive drug use were exploratory. DAR, D-dimer-to-albumin ratio; DVT, deep vein thrombosis; ICU, intensive care unit; OR, odds ratio.

Supplementary Table S4. Availability and descriptive distribution of imaging and echocardiographic findings according to DAR quartiles.

Characteristic	Overall (n = 159)	Q1 (n = 40)	Q2 (n = 40)	Q3 (n = 40)	Q4 (n = 39)
PE-related imaging report with extractable severity features available, n/N (%)	58/159 (36.5)	12/40 (30.0)	9/40 (22.5)	20/40 (50.0)	17/39 (43.6)
Central PE among patients with PE imaging available, n/N (%)	6/58 (10.3)	2/12 (16.7)	1/9 (11.1)	0/20 (0.0)	3/17 (17.6)
Pulmonary infarction among patients with PE imaging available, n/N (%)	2/58 (3.4)	0/12 (0.0)	0/9 (0.0)	1/20 (5.0)	1/17 (5.9)
Echocardiography available, n/N (%)	45/159 (28.3)	8/40 (20.0)	7/40 (17.5)	16/40 (40.0)	14/39 (35.9)
Right heart strain among patients with echocardiography available, n/N (%)	8/45 (17.8)	1/8 (12.5)	0/7 (0.0)	3/16 (18.8)	4/14 (28.6)
Elevated pulmonary artery pressure among patients with echocardiography available, n/N	8/45 (17.8)	1/8 (12.5)	1/7 (14.3)	3/16 (18.8)	3/14 (21.4)

Characteristic	Overall (n = 159)	Q1 (n = 40)	Q2 (n = 40)	Q3 (n = 40)	Q4 (n = 39)
(%)					
PASP available, n/N (%)	25/159 (15.7)	6/40 (15.0)	5/40 (12.5)	7/40 (17.5)	7/39 (17.9)
PASP, mmHg, median (IQR), among available measurements	35.0 (27.0–42.0)	29.5 (25.0–35.5)	35.0 (28.0–37.0)	36.0 (29.5–41.0)	36.0 (28.0–50.0)

Data are presented as n/N (%) or median (IQR). Percentages for imaging and echocardiographic availability were calculated using the full cohort or corresponding DAR-quartile denominators. Percentages for specific imaging and echocardiographic findings were calculated only among patients with the relevant reports available. Patients without relevant reports were classified as having unavailable data rather than as having no abnormality. Given the limited availability and small numbers of positive findings, these data were summarized descriptively without formal between-group comparisons. DAR, D-dimer-to-albumin ratio; IQR, interquartile range; PASP, pulmonary artery systolic pressure; PE, pulmonary embolism.

Supplementary Table S5. Correlations, model performance, internal validation, and formal comparisons of DAR and its component biomarkers for ICU admission

A. Correlations among DAR and its component biomarkers

Comparison	Spearman ρ
log-DAR vs. log-D-dimer	0.995
log-DAR vs. log-albumin	−0.414
log-D-dimer vs. log-albumin	−0.349

B. Apparent model performance for ICU admission

Model	N	Events	AIC	Apparent AUC (95% CI)
Clinical covariates	159	22	137.2	0.568 (0.448–0.687)
Clinical covariates + log-D-dimer	159	22	118.4	0.785 (0.682–0.888)

Model	N	Events	AIC	Apparent AUC (95% CI)
Clinical covariates + log-albumin	159	22	132.6	0.743 (0.638–0.847)
Clinical covariates + log-D-dimer + log-albumin	159	22	119.0	0.792 (0.697–0.887)
Clinical covariates + log-DAR	159	22	117.5	0.788 (0.687–0.889)

C. Bootstrap optimism-corrected performance and calibration

Model	Optimism-corrected AUC	Corrected calibration intercept	Corrected calibration slope
Clinical covariates	0.454	−1.581	0.133
Clinical covariates + log-D-dimer	0.737	−0.333	0.770
Clinical covariates + log-albumin	0.660	−0.761	0.558
Clinical covariates + log-D-dimer + log-albumin	0.738	−0.413	0.723
Clinical covariates + log-DAR	0.738	−0.345	0.762

D. Formal comparisons with the log-D-dimer model

Model compared with log-D-dimer	ΔAUC	DeLong p value	ΔAIC	Likelihood-ratio χ^2	p value
log-D-dimer + log-albumin	+0.008	0.486	+0.6	1.42	0.234
log-DAR	+0.003	0.471	−0.9	NA	NA

All models included the same 159 patients and 22 ICU admission events. Clinical covariates included age, sex, recorded malignancy, and concomitant DVT. Albumin was natural-log-transformed because log-DAR is mathematically equivalent to log-D-dimer minus log-albumin. Apparent AUC confidence

intervals were calculated using the DeLong method. Optimism-corrected AUCs, calibration intercepts, and calibration slopes were estimated using 2000 nonparametric bootstrap resamples. An ideal calibration intercept is 0, and an ideal calibration slope is 1. Changes in AUC were evaluated using paired DeLong tests. The likelihood-ratio test was used only for the nested comparison between the log-D-dimer model and the model additionally containing log-albumin. Changes in AUC and AIC were calculated relative to the log-D-dimer model. A lower AIC indicates better model fit. AIC, Akaike information criterion; AUC, area under the receiver operating characteristic curve; DAR, D-dimer-to-albumin ratio; DVT, deep vein thrombosis; ICU, intensive care unit; NA, not applicable.

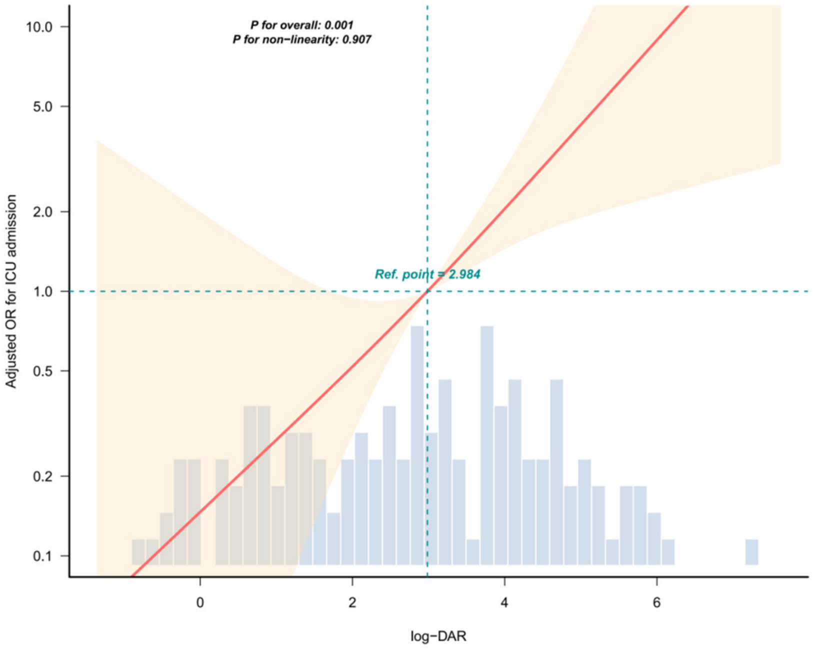

Supplementary Figure S1. Restricted cubic spline analysis of log-DAR and ICU admission. *The model included 159 patients and 22 ICU admission events and was adjusted for age, sex, recorded malignancy, and concomitant DVT. Four knots were placed at the 5th, 35th, 65th, and 95th percentiles of log-DAR (−0.123, 2.151, 3.811, and 5.704), with the median log-DAR value of 2.984 as the reference. The solid line represents the adjusted odds ratio, the shaded area represents the 95% confidence interval, and the histogram shows the distribution of log-DAR. Wald* χ^2 *tests indicated an overall association (P = 0.001) but no evidence of nonlinearity (P = 0.907).*

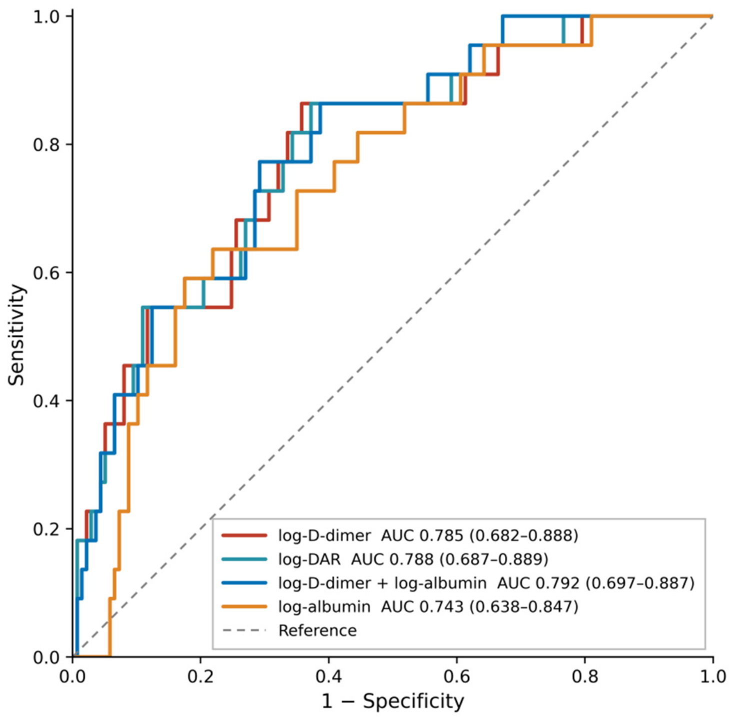

Supplementary Figure S2. ROC curves comparing DAR and its component biomarkers for ICU admission. *Curves are presented for models containing log-D-dimer, log-albumin, log-DAR, or log-D-dimer and log-albumin entered as separate variables. All models were adjusted for age, sex, recorded malignancy, and concomitant DVT. The analysis included 159 patients and 22 ICU admission events. AUC values, 95% confidence intervals, and formal model comparisons are reported in Supplementary Table S5.*
